# Lithium efficacy in bipolar depression with flexible dosing: A six-week, open-label, proof-of-concept study

**DOI:** 10.3892/etm.2014.1864

**Published:** 2014-07-24

**Authors:** RODRIGO MACHADO-VIEIRA, MARCUS V. ZANETTI, RAFAEL T. DE SOUSA, MARCIO G. SOEIRO-DE-SOUZA, RICARDO A. MORENO, GERALDO F. BUSATTO, WAGNER F. GATTAZ

**Affiliations:** 1 Laboratory of Neuroscience LIM-27, Department and Institute of Psychiatry, School of Medicine, University of São Paulo (HC-FMUSP), São Paulo, Brazil; 2 Center for Interdisciplinary Research on Applied Neurosciences (NAPNA), University of São Paulo, São Paulo, Brazil; 3 Mood Disorders Unit (GRUDA), Department and Institute of Psychiatry, School of Medicine, University of São Paulo (HC-FMUSP), São Paulo, Brazil

**Keywords:** bipolar disorder, depression, lithium, treatment, trial

## Abstract

Lithium has a narrow therapeutic index with a subtle balance between effectiveness and adverse effects. Current guidelines recommend the use of lithium as a treatment for acute bipolar depression; however, the therapeutic range for the treatment has not been fully defined. Recently, the adjunctive lower lithium dose in bipolar depression has revealed potential efficacy; however, no study has investigated it predominantly in monotherapy. In this open-label, proof-of-concept study, 31 individuals with bipolar disorder during a depressive episode were randomized and 29 were followed up for six weeks with flexible lithium dosing. All subjects had a 21-item Hamilton Rating Scale for Depression (HAM-D) score of ≥18 at baseline. Subjects were divided into two groups, with higher (Li ≥0.5 mEq/l) or lower (Li <0.5 mEq/l) blood lithium levels. Response and remission rates were evaluated using the HAM-D scores. Following 6 weeks of lithium treatment, the remission rate for all patients was 62.0%. The plasma lithium levels did not impact the clinical response. However, subjects with higher blood lithium levels had an increased prevalence of nausea, restlessness, headaches and cognitive complaints. The results indicate that the lithium dose for the treatment of bipolar depression in an individual should be based on the clinical efficacy and side-effects. In the context of personalized psychiatric treatments, it is necessary to evaluate the therapeutic action of lithium with individual regimens in order to develop more tolerable and effective treatment approaches.

## Introduction

Bipolar disorder (BD) is a chronic and disabling psychiatric disorder, with patients spending up to half of their time in a state of depression (1,2). Thus, pharmacological approaches in BD have focused primarily on the treatment of bipolar depressive episodes. Despite recent advances, only a few agents have proven efficacy in treating bipolar depression, with a wide range of controversial options being tested. Lithium is a primary agent for the treatment of BD in multiple phases worldwide (3). Furthermore, lithium has significant neuroprotective effects (3) and may also, at low doses, decrease the risk of dementia in BD (4).

Current guidelines recommend the use of lithium as a treatment for acute bipolar depression (5,6). Data from the European drug surveillance program reveal that lithium is the most commonly prescribed agent for bipolar depression in combined therapy (33%) (5). Early studies suggested that blood lithium levels >0.8 mEq/l are necessary in maintenance therapy for BD (7). More recently, it was observed that lower plasma lithium levels (0.4–0.6 mEq/l) were associated with higher relapse rates in maintenance therapy (8). However, a recent large study, involving a 24-week follow-up, revealed no association between an improvement in depression or mania ratings in patients with BD and their blood lithium levels [low (<0.4 mEq/l) vs. high (0.4–0.9 mEq/l)] (9). Furthermore, a reanalysis of the study by Gelenberg *et al* (7) demonstrated that patients treated with low levels of lithium during the pre- and post-randomization phases remained stable during the study period (10). These results may encourage a more limited use of higher lithium doses, even in acute mood episodes in BD.

Furthermore, studies investigating the use of lithium in BD treatment have revealed high drop-out rates and morbidity due to side-effects (11) In these studies, the minimum lithium levels were 0.5–0.6 mEq/l (12–14), with drop-out rates reaching 61% (13). In the current six-week, open-label, proof-of-concept study, the efficacy and tolerability of lithium monotherapy with flexible doses in bipolar disorder I (BDI) and II (BDII) depressive episodes were evaluated.

## Materials and methods

### Study design

In the present study, individuals were required to have a DSM-IV-TR diagnosis of BDI or BDII in a current depressive episode, according to the structured clinical interview for DSM-IV axis I disorders (SCID-I) (15). Patients began the lithium treatment on a dose of 450 mg/day and subsequent dosage adjustments were permitted in a flexible manner to a dose of ≤900 mg/day, based on the clinical efficacy (response/remission rate) of individual patients and the level of lithium in their plasma. Measurements for the plasma lithium levels were obtained on days 7 and 14, and at the endpoint. Short-term use of benzodiazepines/hypnotics for a maximum of five consecutive days (once per day) was permitted during the follow-up period. Subjects with BDII had not taken any psychopharmacological treatment for at least six weeks prior to the study while subjects with BDI may have been treated with a maximum of one mood stabilizer or one antipsychotic agent. All patients had not been previously treated with lithium. Lithium was obtained from Eurofarma (São Paulo, Brazil). The current study was a single center study conducted at the Institute of Psychiatry of the University of São Paulo (São Paulo, Brazil), clinical trial number NCT01919892.

### Subjects and outcome measures

Male and female outpatients, aged between 18–45 years were eligible to participate in the study. All subjects were recruited and followed up at the Mood Disorders Program, Laboratory of Neuroscience LIM27, Institute of Psychiatry of the University of São Paulo (São Paulo, Brazil) between August 2010 and June 2012. Clinical assessments included: the score for the 21-item Hamilton Rating Scale for Depression (HAM-D) as the primary outcome, and the Young Mania Rating Scale (YMRS) and the Clinical Global Impression (CGI) scale scores as secondary outcomes. A HAM-D score of ≥18 was also required for inclusion. All adverse effects were recorded using the Udvalg for Kliniske Undersogelse (UKU) side-effects rating scale. Early improvement was defined as ≥20% improvement from the baseline HAM-D score following one week of lithium treatment. The response rate was defined as a reduction of ≥50% in the HAM-D score at endpoint and the remission rate was characterized as a HAM-D score of <8 at endpoint. There was no run-in period. At the onset of the study, all subjects had <3 mood episodes in their lifetime and an illness duration of ≤5 years. Exclusion criteria included: rapid cycling in the past 12 months, previous head trauma, a current Axis I psychiatric disorder other than BD, subjects submitted to electroconvulsive therapy, significantly abnormal laboratory test results and any chronic medical condition. The diagnosis was determined by experienced psychiatrists who held an inter-rater reliability score of >0.9 for HAM-D and YMRS. The first assessment was carried out at baseline [visit (V)1] and subsequent visits for clinical assessment, dose adjustment and monitoring of the levels of lithium were performed at the end of weeks 1 (V2), 2 (V3), 4 (V4) and 6 (V5). The present study was approved by the local institutional ethics committee of the Clinics Hospital (São Paulo, Brazil) and all patients provided written consent prior to participation in the study.

### Statistical analyses

The primary outcome was the degree of change in depressive symptoms (HAM-D scores) analyzed using mixed-effects random regression. The mixed-effects regression was used to analyze changes in HAM-D assessments over the 6 weeks of follow-up and their association with time and clinical variables that were potentially associated with response to lithium treatment including: age, gender, duration of illness, history of psychosis, BD subtype and plasma lithium levels at week 6. Inter-group comparisons of continuous variables with a Gaussian distribution were performed using the Student’s t-test. The analyses of continuous variables with a non-parametric distribution were conducted using the Mann-Whitney U Test (the Kolmogorov-Smirnov test was used to verify the normality of the data within each study group). The chi-square test was used for the comparison of categorical factors. The Pearson coefficient was used to evaluate the correlation between the relative change in HAM-D scores over time and other demographic and clinical variables. Statistical significance was set at P<0.05 (two-tailed) and data are presented as means ± standard deviations. All statistical analyses were carried out using SPSS software, version 16.0 (SPSS, Inc., Chicago, IL, USA).

## Results

### Patient characteristics

A total of 42 patients were accessed for eligibility to participate in the current study following a telephone screening at the Mood Disorders Program, LIM27, University of São Paulo. Of these, 31 patients were included in the present study. Two drop-outs were observed: one patient left due to personal problems unrelated to the study and the other patient was excluded due to noncompliance to the treatment (undetectable blood lithium levels). Thus, the final sample at endpoint comprised of 29 acutely depressed bipolar patients.

Table I summarizes the clinical and demographic data for the subgroups of patients with BDI and BDII. The final study group comprised 21 females (72.4%). The patients had a mean age of 28.4±5.4 years and a diagnosis of BDI (n=11; 37.9%) or BDII (n=18; 62.1%). At baseline, the patients had a mean duration of illness of 36.1±19.6 months and presented mean HAM-D and YMRS scores of 22.5±3.5 and 6.1±5.6, respectively. Out of the 29 patients that completed the current study, 21 were naïve to lithium treatment (72.4%) and 25 (86.2%) had been medication-free for at least six weeks prior to participating in the study. Three of the patients had YMRS scores >8. Furthermore, only four (13.8%) patients had a previous history of psychotic symptoms.

The 29 patients who completed the study were followed-up with lithium monotherapy (n=25) or lithium therapy associated with another mood stabilizer or antipsychotics (n=4). The mean plasma level of lithium at V5 was 0.49±0.20 mEq/l.

### Clinical assessment scores

**Figure 1:**
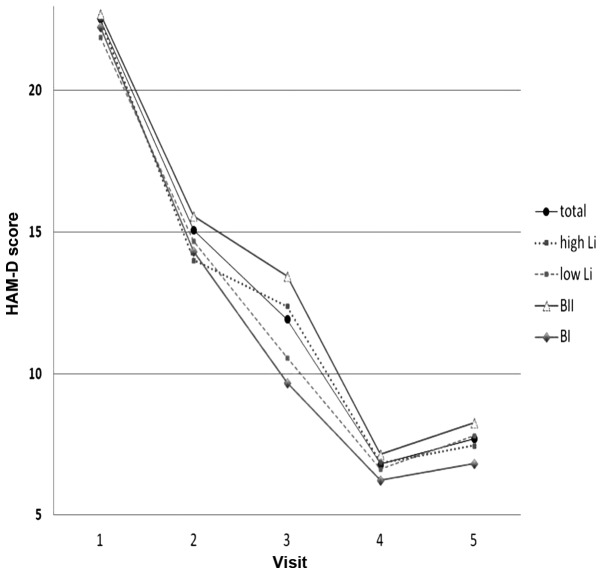
Total Hamilton Rating Scale for Depression (HAM-D) scores during the six-week follow-up period: baseline (visit 1) and end of weeks 1 (visit 2), 2 (visit 3), 4 (visit 4) and 6 (visit 5). No significant difference was observed when comparing low (<0.5 mEq/l) and high (>0.5 mEq/l) blood lithium levels with regards to antidepressant efficacy. The diagnosis of bipolar disorder I (BI) or bipolar disorder II (BII) did not significantly influence the clinical outcome (P>0.05).

A significant reduction in HAM-D scores was observed over the six weeks of follow-up in the whole group of BD patients (P<0.001). Following six weeks of lithium treatment, the response and remission rates in the total study population were 86.2% and 62.0%, respectively. The mean HAM-D and YMRS scores following six weeks of lithium treatment were 7.3±5.9 and 3.8±8.6, respectively. When comparing BDI and BDII, similar remission rates were observed at endpoint; 63.6 vs. 61.1%, respectively (Fig. 1). The CGI scores, which were 4.21±0.55 pretreatment, were significantly decreased at endpoint (2.14±0.99; P<0.001) with no significant difference identified between the patients with BI and those with BII (P=0.86). None of the patients experienced a switch to (hypo)mania during the follow-up period.

### Plasma lithium levels

At week 6, 15 patients had lower (Li<0.5 mEq/l) and 14 presented higher (Li≥0.5 mEq/l) plasma lithium levels. The plasma lithium levels did not differentially impact the number of patients who responded (n=13 responders with lower levels vs. n=12 responders with higher levels; P=0.94). Furthermore, the use of hypnotics (n=7), if necessary, was not observed to influence the clinical outcome (HAM-D scores; P=0.37). No other significant association was observed in the mixed-effects regression. No significant differences were observed in patients in remission (n=18) vs. those not in remission (n=11) at endpoint (a HAM-D score <8) with regard to oral dosage, blood lithium levels, gender and bipolar disorder subtype (data not shown). No association was observed between HAM-D scores and lithium dose (P=0.99) or blood levels (P=0.87) at endpoint.

### Response rate and side-effects of lithium treatment

With regard to the potential predictors of response rate, only a previous history of psychosis was observed to be significantly associated with a faster reduction in HAM-D scores in response to lithium treatment (F=3.68, P=0.008). None of the patients experienced a switch to (hypo)mania during the follow-up period. Baseline YMRS scores did not influence the response rate (data not shown). Common adverse effects observed were polydipsia/polyuria (62.1%), cognitive complaints (41.4%), nausea (31.0%), increased oniric activity (31.0%) and sedation (31.0%). In subjects with higher lithium levels compared with those with lower levels, an increased prevalence of nausea (50.0 vs. 13.3%; P=0.03), restlessness (42.9 vs. 6.7%; P=0.02) and headaches (42.9 vs. 6.7%; P=0.02) was observed. No patient drop-outs due to side-effects occurred during the follow-up period.

## Discussion

In the present six-week, open-label, single-arm study, lithium revealed a significant antidepressant efficacy in short-term BD. No differences in efficacy were observed with lower levels (<0.5 mEq/l) vs. standard levels (0.5–1.2 mEq/l) of lithium. However, the current study population comprised predominantly young subjects with a shorter duration of illness and included predominantly BDII cases, which may have had an influence on the positive clinical outcome.

The similarity in the efficacy of standard vs. ‘sub-therapeutic’ lithium levels that was observed in the BD treatment in the present study has been indirectly addressed by recent studies on BD (9,16). The LiTMUS study evaluated symptomatic BD; however, unlike the current study it was solely based on the use of lithium monotherapy (9). A study by Perlis *et al* did not identify a superior efficacy of using higher lithium levels (≥0.8 mm/l) in long-term maintenance therapy (10). Furthermore, a higher prevalence of nausea, restlessness, headaches and cognitive complaints was identified in BD subjects with higher blood lithium levels in the present study. Other studies have experienced potential compliance issues with patients with higher blood lithium levels. In a study by Amsterdam and Shults, only 37.5% of subjects under higher lithium doses completed a 12-week trial with lithium monotherapy in bipolar depression compared with a completion rate of 79.1% for patients treated with antidepressant (12). Similarly, a previous study on bipolar depression described the early termination of 61% (30 out of 49) of patients with expected lithium levels of 0.8–1.2 Meq/l (13). The limitations of the current study include the lack of a placebo group, the open-label design and the relatively small sample size.

The current study aimed to define the optimal lithium dose for BD treatment based on the clinical responses of patients and their tolerance of lithium. The results revealed that lower lithium doses demonstrated similar improvements to higher doses; however, they were associated with lower drop-out rates due to side-effects. In the context of personalized psychiatric treatments, it is critical to evaluate the therapeutic action of lithium with individual regimens in order to develop more effective and tolerable approaches to BD treatment. Further studies are required to investigate this.

## Figures and Tables

**Table I:** Demographic and clinical information of subjects with bipolar I and II disorders.

Characteristics	Bipolar I (n=11)	Bipolar II (n=18)	P-value
Age (years)	29.1±6.3	27.9±5.	0.592
Female gender	9 (81.8)	12 (66.7)	0.376
Duration of illness (months)	43.3±20.3	31.6±18.3	0.121
History of psychosis	4 (36.4)	0 (0%)	0.006
Naïve to treatment at week 0	6 (54.5)	15 (83.3)	0.092
Medication-free at week 0	8 (72.7)	17 (94.4)	0.100
HAM-D score at baseline	22.2±4.5	22.8±2.9	0.669
Ham-D score at week 6	7.5±5.7	7.1±6.1	0.852
YMRS score at baseline	6.4±6.3	5.9±5.3	0.982
YMRS score at week 6	5.2±13.2	2.9±4.0	0.912
Response rate at week 6	8 (72.7)	17 (94.4)	0.100
Remission rate at week 6	7 (63.6)	11 (61.1)	0.892
Levels of lithium at week 6 (mmol/l)	0.51±0.21	0.48±0.19	0.729

Data are presented as mean ± standard deviation or as a number with the percentage in parentheses. HAM-D, 21-item Hamilton Rating Scale for Depression; YMRS, Young Mania Rating Scale.
